# Factors associated with bronchiectasis in patients with moderate–severe chronic obstructive pulmonary disease

**DOI:** 10.1097/MD.0000000000004219

**Published:** 2016-07-22

**Authors:** Jianmin Jin, Wenling Yu, Shuling Li, Lijin Lu, Xiaofang Liu, Yongchang Sun

**Affiliations:** a Department of Respiratory Medicine; b Department of Radiology, Beijing Tongren Hospital, Capital Medical University, Dongjiaominxiang, Dongcheng District; c Department of Respiratory and Critical Care Medicine, Peking University Third Hospital, Huayuanbeilu, Haidian District, Beijing, China.

**Keywords:** Immunoglobulin E, chronic obstructive pulmonary disease, bronchiectasis, risk factor

## Abstract

A high prevalence of bronchiectasis was found by chest computed tomography (CT) in patients with moderate–severe chronic obstructive pulmonary disease (COPD), and it was shown to be associated with more severe symptoms, higher frequency of exacerbations and mortality. The risk factors for bronchiectasis in COPD are not yet clarified.

High-resolution computed tomography (HRCT) of chest was performed in patients with moderate–severe COPD, and the presence and the extent of bronchiectasis were evaluated by two radiologists. Demographic data, respiratory symptoms, lung function, previous pulmonary tuberculosis, serum inflammatory markers, serum total immunoglobulin E (T-IgE), and sputum culture of *Pseudomonas aeruginosa* were compared between those with and without bronchiectasis. Multivariate logistic regression analysis was used to determine the independent factors associated with bronchiectasis.

We enrolled 190 patients with stable COPD, of which 87 (87/190, 45.8%) had bronchiectasis on HRCT. Compared with those without bronchiectasis, COPD patients with bronchiectasis were more likely to be males (*P* = 0.021), had a lower body mass index (BMI) (*P* = 0.019), a higher prevalence of previous tuberculosis (*P* = 0.005), longer history of dyspnea (*P* < 0.001), more severe dyspnea (*P* = 0.041), higher frequency of acute exacerbation (*P* = 0.002), higher serum concentrations of C-reactive protein (CRP) (*P* = 0.017), fibrinogen (*P* = 0.016), and T-IgE [*P* = 0.004; for log_10_(T-IgE), *P* <0.001]. COPD patients with bronchiectasis also showed poorer lung function (for FEV_1_/FVC, *P* = 0.013; for FEV_1_%predicted, *P* = 0.012; for global initiative for chronic obstructive lung disease (GOLD) grades, *P* = 0.035), and a higher positive rate of sputum *P aeruginosa* (*P* = 0.020). Logistic regression analysis demonstrated that male gender (*P* = 0.021), previous tuberculosis (*P* = 0.021), and increased level of serum T-IgE [for log_10_(T-IgE), *P* < 0.001] were risk factors for coexistent bronchiectasis. More notably, the level of serum T-IgE [log_10_(T-IgE)] was positively correlated with the extent of bronchiectasis in COPD patients (*r* = 0.208, *P* = 0.05).

Higher serum T-IgE, male gender, and previous tuberculosis are independent risk factors for coexistent bronchiectasis in COPD. The association of T-IgE with the extent of bronchiectasis also suggests that further investigations are needed to explore the potential role of IgE in the pathogenesis of bronchiectasis in COPD.

## 1 Introduction

Chronic obstructive pulmonary disease (COPD) is a heterogeneous disorder characterized by persistent airflow limitation associated with chronic airway inflammation and emphysema. Besides smoking, factors such as age, gender, bronchial hyperreactivity, and tuberculosis are also risk factors for disease development and progression of COPD.^[1]^ With increasing use of high-resolution computed tomography (HRCT) in the assessment of COPD, a high prevalence of bronchiectasis was found among COPD patients, especially those with moderate–severe disease. The existence of bronchiectasis was shown to be associated with more severe symptoms, higher frequency of exacerbations and mortality, by several studies.^[2–5]^ Therefore, bronchiectasis has been proposed as one of the comorbidities of COPD by global initiative for chronic obstructive lung disease (GOLD) 2014.^[1]^ The etiology of noncystic fibrosis bronchiectasis is mostly associated with pulmonary infection (viral, bacterial, Mycobacterial, or fungal) and chronic inflammation of bronchi, although other factors such as malnutrition and extremes of age are also contributory to its occurrence.^[6,7]^ Until now, the causes and mechanisms of bronchiectasis in COPD are not clear. Understanding of the risk factors for bronchiectasis in COPD should provide insights for further investigation to the pathogenesis of this important comorbidity. Therefore, we set out to find the risk factors for bronchiectasis in a well-defined cohort of patients with stable moderate–severe COPD. Our results show that male gender, previous tuberculosis, and most remarkably, serum total immunoglobulin E (T-IgE) are independent risk factors for coexistent bronchiectasis in COPD.

## 2 Subjects and methods

### 2.1 Study subjects

Patients with stable COPD visiting Beijing Tongren Hospital, Capital Medical University from July 2008 to July 2014 were evaluated. The patients were diagnosed to have COPD by the following criteria, as described in our previous study:^[8]^ (i) age >40 years, (ii) history of smoking (smoking index >10 pack-year) and/or exposure to noxious dusts/chemical agents/biomass fuel (mostly coal or wood burning stoves) for >10 years, (iii) chronic cough and/or wheeze for >3 months in each year for two consecutive years, and (iv) irreversible obstructive dysfunction defined by postbronchodilator FEV_1_/FVC < 70% on spirometry. The enrolled patients showed no evidence of parasite infection and no history of food allergy and doctor-diagnosed allergy such as eczema and allergic rhinitis.^[8]^ Patients were excluded from the study if they met any of the following criteria as we previously described: receiving therapy of systemic corticosteroid in the preceding 8 weeks; receiving any other therapy of immunosuppression; with allergic bronchopulmonary mycosis, interstitial lung diseases, active pulmonary tuberculosis, autoimmune diseases, and severe heart failure; previous pulmonary tuberculosis resulting in severe lung damage (“destroyed lung”); doctor-diagnosed bronchiectasis or asthma before diagnosis of COPD; and history of measles or whooping cough during childhood.

The study was approved by the local ethics committee of Beijing Tongren Hospital, Capital Medical University, and written informed consent was obtained from all of the patients.

### 2.2 Lung HRCT and evaluation of bronchiectasis and emphysema

HRCT of the chest was performed using a 64-row, multiple-detector CT scanner (Philips Company, the Netherlands). The presence and extent of bronchiectasis (Smith score) and severity of emphysema (Goddard score) were evaluated by 2 radiologists experienced in the interpretation of HRCT and blinded to the patients’ clinical data. The radiologists finished their evaluation independently and the differences in the reading were resolved by their final consensus.

The diagnosis of bronchiectasis was made if chest HRCT showed bronchial wall thickening with the ratio of the diameter of bronchus to that of the accompanying pulmonary artery being >1.1 (signet ring sign) or the lack of tapering of bronchi (tramline sign). The extent of bronchiectasis was scored for each pulmonary lobe, with the lingula as a separate lobe. Mild bronchiectasis only visible in a single pulmonary segment was not counted, as this may exist in a significant percentage of healthy population.^[9]^ The grading system proposed by Smith and coworkers^[3]^ were adopted in our study; the absence of bronchiectasis being scored as 0, bronchiectasis in fewer than 25% of bronchi as 1, in 25% to 49% of bronchi as 2, in 50% to 74% as 3, and in 75% or more as 4, as reported by others.^[9]^ The total score ranged from 0 to 24 points. Patients with a score ≤1 were considered as normal. The type of bronchiectasis (cylindrical, cystic, or mixed) was defined according to the morphology of bronchiectasis.

The severity of emphysema was visually assessed with the modified Goddard scoring system.^[10]^ Six images were analyzed in three slices (including the aortic arch, carina, and 1–2 cm above the highest hemidiaphragm) of both lungs, and a total score of all images was considered as a representative value of the severity of emphysema for each patient. Each image was classified as normal (score 0), 5% affected (score 0.5), 25% affected (score 1), 50% affected (score 2), 75% affected (score 3), and >75% affected (score 4), and so the total score of each subject may range from a minimum of 0 to a maximum of 24.

### 2.3 Pulmonary function test

Spirometry (JAEGER, MasterScreen-body + diffusion + APS, Germany) was performed to determine the lung function measurements and bronchodilator reversibility. Post-bronchodilator FEV_1/_FVC% and FEV_1_ were measured 15 minutes after inhalation of 400 μg salbutamol.

### 2.4 Determination of previous infection of tuberculosis

Previous tuberculosis was defined as present if the patients met one of the following criteria: a previous history of doctor-diagnosed pulmonary tuberculosis and the patient had been given anti-tuberculosis treatment; the presence of discrete linear or reticular fibrotic scars, or dense nodules with distinct margins within the upper lobes on CT scan, with calcification of the lesions and/or local lymph nodes.^[11–13]^

### 2.5 Definition of respiratory symptoms and exacerbations

Chronic cough and expectoration were identified as present if the symptoms lasted for ≥3 consecutive months.^[8]^ Exertional dyspnea was defined as present if the mMRC (Modified Medical Research Council Questionnaire) score was equal to or >1. The duration of a symptom such as dyspnea was defined as the time length from its onset to enrollment of patient in the study.^[8]^ Frequency of acute exacerbation (AE) was defined as the frequency of hospitalization or emergency visits because of AE during the year before enrollment.

### 2.6 Detection of serum IgE and fungus-specific IgE

An automatic immunoassay system (ImmunoCap TM 100, Pharmacia Company, Sweden) was adopted according to the manufacturer's instructions. As we previously described,^[8]^ the lower limit of detection (LLD) of serum T-IgE, and fungus-specific IgE (including IgE specific to *Aspergillus fumigatus*, *Penicillium chrysogenum,* and *Candida albicans*) is 2 kU/L and 0.01 kUA/L, respectively. Levels of serum T-IgE >60 kU/L, and fungus-specific IgE >0.35 kUA/L were defined as abnormally high.

### 2.7 Bacterial culture of sputum samples

Results of sputum cultures were reviewed retrospectively and positive cultures for *P aeruginosa* were compared between groups. The sputum samples were obtained when the patients had experienced a severe exacerbation. A qualified sputum sample was defined as having fewer than 10 squamous epithelial cells and >25 leukocytes per low-powered field.^[2,4]^

### 2.8 Statistical analysis

The statistical package SPSS version 17.0 (SPSS, Chicago, IL) was adopted for statistical analysis as previously described.^[8]^ Data were expressed as mean ± SD (standard deviation). *t* Test (for normal distribution parameters) and Mann–Whitney *U* test (for abnormal distribution parameters) were used for comparisons of continuous data between different groups. Categorical variables were analyzed by χ^2^ test, and Spearman Correlations were used for correlation analysis.^[8]^ Risk factors for bronchiectasis were analyzed with multivariate logistic regression analysis. *P* values ≤0.05 were considered significant in this study.^[5]^

## 3 Results

### 3.1 Clinical characteristics of subjects with COPD

The clinical data of 190 patients with COPD were shown in Table 1. There were 121 males (63.6%) and 69 females (36.4%) with a mean age of 78 years, and the majority of the patients were smokers (81.0%). The GOLD spirometry classification ranged from 2 to 4, with 67 patients (35.2%) in GOLD 2, 87 (45.8%) in GOLD 3, and 36 (19.0%) in GOLD 4.

**Table 1:**
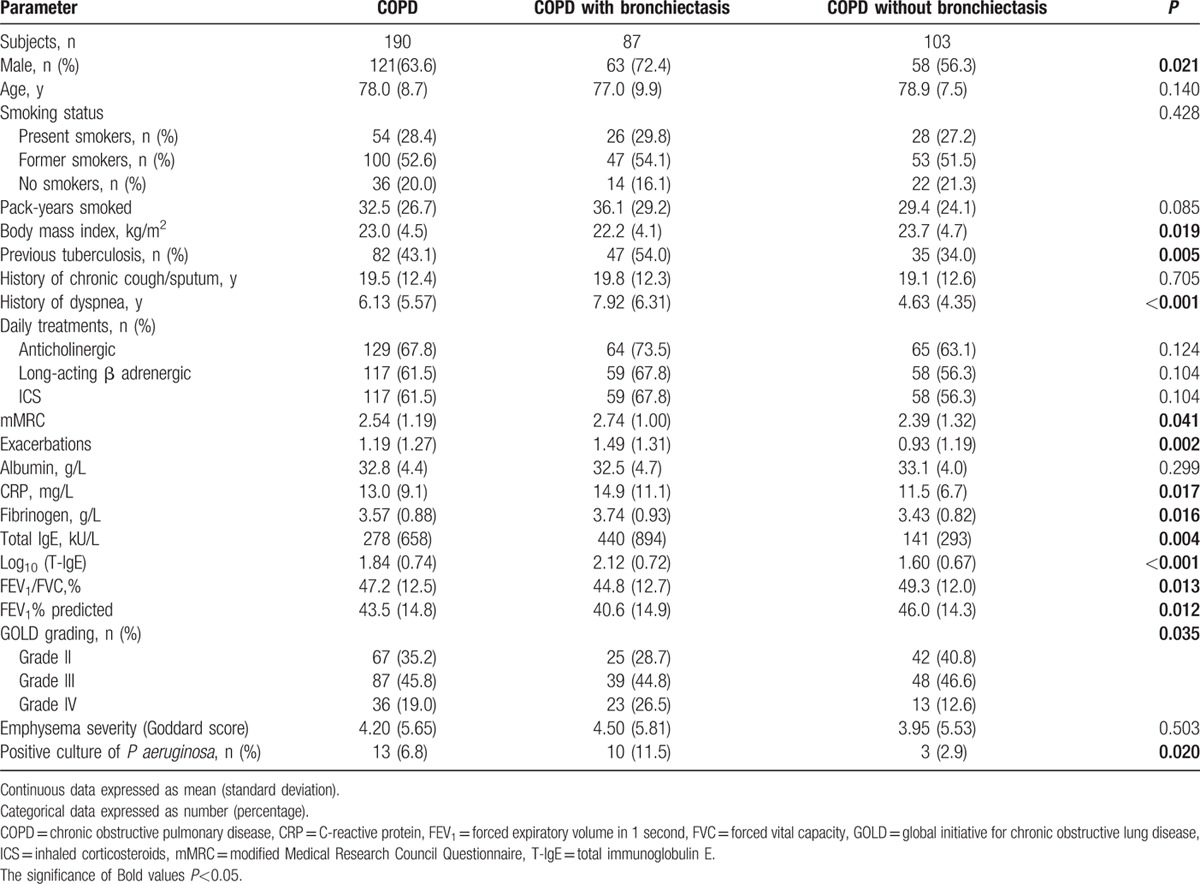
Baseline and clinical characteristics of subjects with COPD, with and without bronchiectasis.

Eighty-two patients (82/190, 43.1%) had previous tuberculosis, of which 35 (35/190, 18.4%) had received anti-tuberculosis therapy, and the remaining 47 (47/190, 24.7%) had only CT signs of inactive tuberculosis. As for the symptoms, the average time course of chronic cough/expectoration and that of exertional dyspnea were 19.5 years and 6.13 years respectively. Serum T-IgE levels varied significantly among different subjects, but the variation of its numerical value [log_10_(T-IgE)] decreased remarkably, the average of which was 1.84. *Pseudomonas aeruginosa* was found positive in sputum samples from 13 cases (13/190, 6.8%).

Emphysema was identified on HRCT in 122 patients (122/190, 64.2%), and the Goddard score was 4.20 on average. Bronchiectasis was found on HRCT in 87 cases (87/190, 45.8%), with the Smith score ranging from 2 to 22 (5.99 ± 4.74). The majority of the patients (82/87, 94.3%) showed cylindrical bronchiectasis, and mixed bronchiectasis was found in the other 5 patients (5/87, 5.7%).

### 3.2 Characteristics of COPD patients with and without bronchiectasis

Comparison of baseline and clinical data between COPD with and without bronchiectasis was shown in Table 1. There was no significant difference in age, smoking status, history of chronic cough/expectoration, and maintenance medication. COPD patients with bronchiectasis were more likely to be males (72.4% vs 56.3%, *P* < 0.05), had a lower body mass index (BMI, 22.2 vs 23.7, *P* < 0.05), and a higher prevalence of previous tuberculosis (54% vs 34%, *P* < 0.01). In addition, COPD patients with bronchiectasis had a longer history of exertional dyspnea (7.92 vs 4.63, *P* < 0.001), more severe dyspnea (mMRC score, 2.74 vs 2.39, *P* < 0.05), and higher frequency of AEs in the previous year (1.49 vs 0.93, *P* < 0.01). Systemic inflammatory markers, including CRP and fibrinogen, were significantly elevated in COPD patients with bronchiectasis (14.9 vs 11.5, 3.74 vs 3.43, respectively, *P* < 0.05 for both). Interestingly, the serum level of T-IgE and its numerical value were significantly higher in COPD patients with bronchiectasis as compared to those without it (440 vs141, *P* < 0.01; 2.12 vs1.60, *P* < 0.001). Moreover, COPD patients with bronchiectasis showed a worse pulmonary function, more severe GOLD grading, and higher positive rate of *P aeruginosa*. However, there was no significant difference in emphysema severity (Goddard score) between the two groups.

### 3.3 Risk factors associated with the presence of bronchiectasis in COPD patients

Potential associated factors, including sex, previous tuberculosis, positive sputum *P aeruginosa*, BMI, mMRC score, the frequency of AE, GOLD grading, FEV_1_/FVC%, FEV_1_%predicted, serum T-IgE level, Log_10_(T-IgE), serum concentrations of CRP and fibrinogen, were taken as the independent variables in a multivariate logistic regression analysis. Our result showed that male gender, previous tuberculosis, and increased level of serum T-IgE were the independent risk factors for the coexistence of bronchiectasis in COPD patients (Table 2).

**Table 2:**
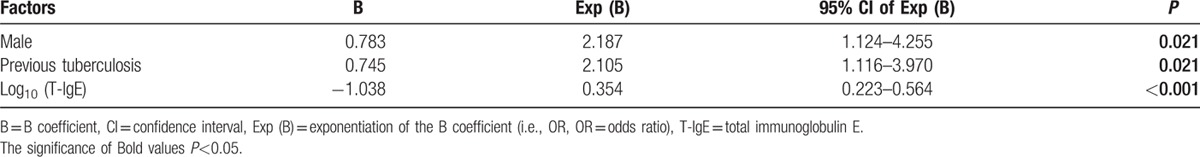
Factors associated with the presence of bronchiectasis in a logistic regression model.

### 3.4 Factors associated with the extent of bronchiectasis in COPD patients

Previous tuberculosis, sex, and positive culture of *P aeruginosa* were firstly suspected to be associated with the extent of bronchiectasis, and therefore COPD patients with bronchiectasis (n = 87) were divided into different groups based on these factors, and the Smith scores were compared respectively. Our result showed that the Smith score of patients with a positive culture of *P aeruginosa* was significantly higher than that of patients with a negative culture (7.90 vs 5.73, *P* < 0.05). However, no significant difference was found in the Smith score either between males and females (6.06 vs 5.78, *P* > 0.05), or between patients with and without previous tuberculosis (5.96 vs 6.01, *P* > 0.05) (Table 3).

**Table 3:**
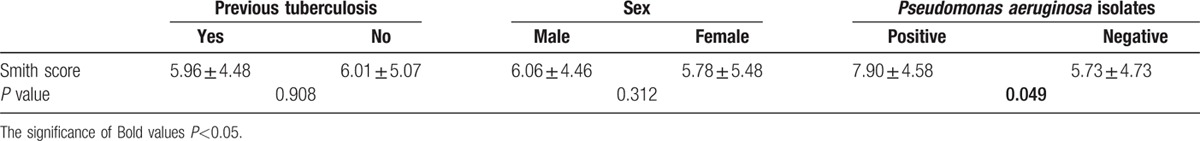
Comparison of Smith score between different groups.

**Figure 1:**
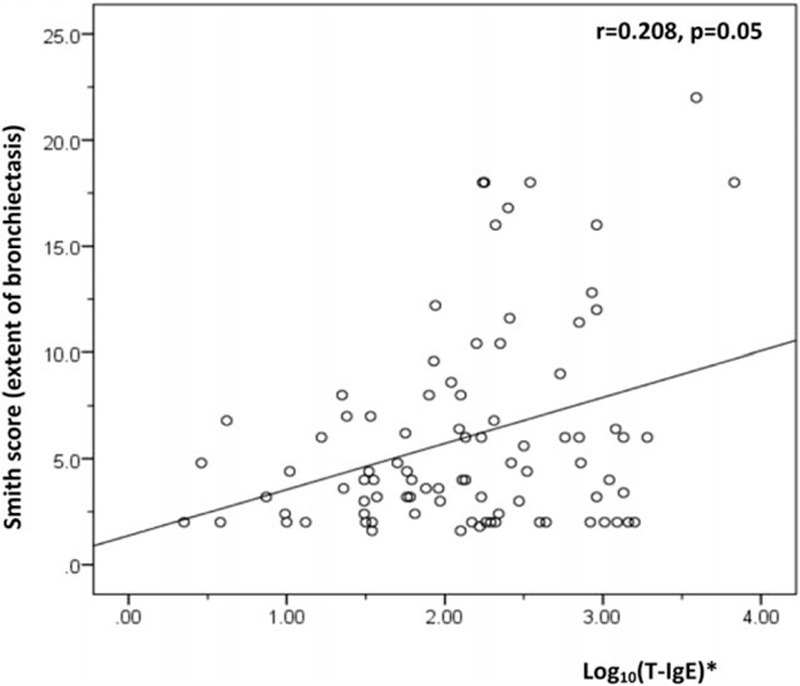
Relationship between T-IgE and extent of bronchiectasis. ^∗^Correlation between log_10_(T-IgE) and extent of bronchiectasis. T-IgE = total immunoglobulin E.

We further used correlation analysis to investigate the factors associated with the extent of bronchiectasis in COPD patients (n = 87). Unexpectedly, the serum T-IgE level showed a positive correlation with bronchiectasis extent (*r* = 0.208, *P* = 0.05) (Fig. 1). However, no correlation was found between the extent of bronchiectasis with age (*r* = −0.088, *P* = 0.416), smoking index (*r* = −0.049, *P* = 0.652), BMI (*r* = −0.055, *P* = 0.612), the time course of chronic cough/expectoration (*r* = –0.063, *P* = 0.559), the time course of dyspnea (*r* = 0.197, *P* = 0.067), mMRC score (*r* = 0.127, *P* = 0.24), frequency of AE (*r* = 0.168, *P* = 0.121), FEV_1_/FVC (*r* = 0.035, *P* = 0.745), FEV_1_%predicted (*r* = −0.016, *P* = 0.88), CRP (*r* = 0.165, *P* = 0.127), fibrinogen (*r* = 0.168, *P* = 0.121), and severity of emphysema (Goddard score) (*r* = 0.035, *P* = 0.745).

## 4 Discussion

Accumulating evidence has revealed the significant association of coexistent bronchiectasis with morbidity and mortality of COPD. However, data on the risk factors for occurrence and severity of bronchiectasis in COPD are scarce. Here we show, in a well-defined cohort of moderate–severe COPD patients, that male gender, previous tuberculosis, and increased level of serum T-IgE are independent risk factors for coexistence of bronchiectasis in COPD. Further analysis reveals that the serum level of T-IgE is correlated with the extent of bronchiectasis. To the best of our knowledge, this is the first study to demonstrate an association of higher serum T-IgE with the presence and severity of bronchiectasis in moderate–severe COPD, which implies that newer approaches targeting IgE production or action (e.g., anti-IgE antibodies) might be of therapeutic potential in attenuating symptoms and outcomes associated with bronchiectasis in a subset of patients with COPD.

The association of serum T-IgE with bronchiectasis in COPD is a remarkable finding of our study and therefore needs more elaboration. Our previous study^[8]^ reported increased serum level of T-IgE in a considerable proportion of COPD patients without obvious atopic disorders. These patients had a longer history of exertional dyspnea, and a poorer lung function.^[8]^ But whether and how IgE plays a role in COPD remain elusive. Recently, a study by Stoll and coworkers^[14]^ found that patients with COPD displayed an overexpression of the high-affinity IgE receptor (FcεRI) on plasmacytoid dendritic cells (pDCs), which was correlated with lung function and GOLD grades. They also found that there was a positive correlation between serum concentration of T-IgE and the FcεRI expression on pDCs. These results provide evidence to support that IgE may be involved in the pathogenesis of some phenotypes of COPD. Cross-linking of FcεRIα impedes the capacity of pDCs to release interferon (IFN)-I and IFN-III, resulting in an impaired antiviral response and an abnormal repair process of airway structural cells,^[15]^ which might be contributory to the formation of bronchiectasis.

In our present study, although clinically apparent atopic diseases such as asthma and allergic rhinitis were carefully excluded, it is still likely that the elevation of serum T-IgE is associated with hypersensitive reactions of the lower airways to irritating antigens. The association of increased IgE with bronchiectasis is well known in several clinical scenarios, for example, allergic bronchopulmonary aspergillosis (ABPA). Interestingly, bronchiectasis was also found in patients with high serum IgE but without ABPA, suggesting that an elevated IgE level may be associated with increased susceptibility to bronchial infections, which in turn result in bronchiectasis.^[16,17]^ Studies have shown that elevated IgE levels are associated with a lack of type 1 T helper (T_H_) lymphocyte activity and shift to T_H_2 responses in both ABPA and hyper-IgE syndrome,^[18,19]^ which may be the underlying mechanisms for recurrent infections and occurrence of bronchiectasis in these conditions.^[17]^ However, further studies focusing on systemic/local imbalance of T_H_1/T_H_2 responses and its association with bronchiectasis in COPD are needed to reveal the potential mechanisms.

Studies in asthma have confirmed that IgE is positively correlated with airway inflammation and remodeling, and its role in asthma is generally believed to be pleiotropic.^[20–23]^ Studies with animal models demonstrated that the contact between airway epithelial cells and airborne allergens induced synthesis of IgE by increased production of IL-4 from lung cells, and IgE could further result in bronchitis and peribronchitis.^[24–26]^ Studies by Vroling et al^[27]^ and Tsai et al^[28]^ indicate that the effect of allergy on airway inflammation in COPD is because of locally enhanced inflammation. However, whether IgE plays an active role in bronchiectasis, or just a marker of intensity of airway inflammation in bronchiectasis, is still speculative.

Pulmonary tuberculosis has been a common cause of bronchiectasis, particularly in tuberculosis high-burdened countries.^[6,29,30]^ A population survey in China (n = 66,546)^[31]^ demonstrated that the prevalence of tuberculosis infection was about 44.5% (29,557/66,546) when a positive response to purified protein derivative (PPD)-RT23 (≥6 mm) was used as the diagnostic criterion. More recently, a prospective cohort study of rural residents in China (n = 21,022)^[32]^ showed that the rate of latent tuberculosis infection, determined by a positive result of IFN-γ release assay, was 19%. Interestingly, there is also a high prevalence of pulmonary tuberculosis in COPD patients,^[30,33,34]^ and tuberculosis is defined as a risk factor for COPD by GOLD.^[1]^ A study by Mao et al^[2]^ showed that the proportion of patients with previous lung tuberculosis defined by a medical history was 12.1% in a Chinese cohort of COPD (n = 896). However, another population survey (age ≥ 50 years, n = 8066) in China^[11]^ demonstrated that the proportion of people with previous lung tuberculosis was 24.2% when the diagnosis was made based on the manifestation of “inactive tuberculosis” on chest radiograph. Most recently, a study from South Africa by Allwood et al^[35]^ demonstrated that in a tuberculosis prevalent population, both questionnaire and chest x-ray (CXR) markedly underestimated the prevalence of previous tuberculosis (36.4% and 43.3%, respectively) in patients with COPD, because lesions compatible with previous tuberculosis were identified on chest CT in 68.3% (71/104) of the subjects. Therefore, besides medical history, typical manifestations of inactive tuberculosis on CT were also used as the diagnostic criterion of previous tuberculosis in our study. Our results showed that the prevalence of previous tuberculosis was 43% in patients with moderate–severe COPD, which was much higher than that previously reported,^[2,4,11]^ but consistent with the findings by Allwood and coworkers.^[35]^ Taken together, these findings suggest that previous tuberculosis of the lung may be an important cause of coexistent bronchiectasis in COPD, and previous tuberculosis should be incorporated into the assessment of COPD, particularly in tuberculosis prevalent countries.

Several studies^[4,5,36,37]^ demonstrated that gender seemed to have an effect on bronchiectasis occurrence in patients with COPD, and recently, a meta-analysis^[38]^ also showed that COPD patients with bronchiectasis were mostly males. In our study, male gender was shown to be a risk factor for bronchiectasis in patients with moderate–severe COPD. Ni and coworkers^[38]^ suggested that higher smoking rate in males could explain this phenomenon. However, no significant effect of smoking status was found on the presence and extent of bronchiectasis in our study. Emerging data suggest that sex hormones may play a role in the pathogenesis of chronic airway diseases.^[39]^ Androgens were found to attenuate allergic inflammation both in mice and in asthmatic patients.^[40,41]^ Decreased levels of testosterone were not only found to be common in males with COPD,^[42]^ but also associated with prognostic indices and inflammation severity in AEs of COPD.^[43]^ Further studies are needed to clarify whether levels of sex hormones are associated with the existence of bronchiectasis in COPD.

In the present study, COPD patients with bronchiectasis also showed a worse nutritional status, longer disease courses, more respiratory symptoms, higher frequency of AEs, more significant inflammatory response, poorer lung function, and a higher positive rate of sputum *P aeruginosa*, which were in agreement with previous reports.^[2,4]^ Our results support the proposition that COPD with bronchiectasis is a clinically relevant phenotype, which may need specific therapies in addition to usual COPD strategies.

Because of the cross-sectional design, our study has several limitations. First, whether the serum level of T-IgE would change was not addressed, although allergic sensitization in adults is generally believed to be stable over time.^[44]^ Second, recall bias may exist since evaluation of the frequency of AE was retrospective. Third, some assessments such as the Body-mass index, airflow Obstruction, Dyspnea, and Exercise (BODE) index and the COPD assessment test (CAT) score were not performed, and the severity of bronchiectasis (Bahalla score) and bronchial wall thickness were not evaluated.

## 5 Conclusions

In summary, our study identified male gender, previous tuberculosis, and increased level of serum T-IgE as independent risk factors for bronchiectasis in patients with moderate–severe COPD. Moreover, the concentration of serum T-IgE was positively correlated with the extent of bronchiectasis, which implied a possibly important role of IgE in bronchiectasis development in COPD. Prospective studies with large sample sizes are needed for evaluating the longitudinal changes of IgE and its effect on the natural course of the disease. Whether modulation of IgE is clinically beneficial for patients with concurrent COPD and bronchiectasis also awaits investigation.
